# Supplementary material for: Unveiling the Role of SlRNC1 in Chloroplast Development and Global Gene Regulation in Tomato Plants
Source: Int J Mol Sci. 2024 Jun 24;25(13):6898. doi: 10.3390/ijms25136898 (PMC11241334; doi:10.3390/ijms25136898)
Supplement: Supplementary file 1 [file ijms-25-06898-s001.zip › ijms-3039279-supplementary.pdf]

Table S1 Primer Sequences

Primers	Sequences (5'-3')
186-GFP-RNC1-F	CgACgACAAgACCgTCACCATGGATCCAAAACCAGATCCTAATTC
186-GFP-RNC1-R	gAggAgAagAgCCgTCgTACCGCCTTAGGTTGGATTTGC
Vigs-RNC1-F	CgACgACAAgACCgTCACCATGGATCCAAAACCAGATCC
Vigs-RNC1-R	gAggAgAagAgCCgTCgATTGGAACTTTTTACATGGGTTT
qPCR-Solyc01g005230-F	GTGCCAGTGTATTTTCCCTC
qPCR-Solyc01g005230-R	TCTTCTGTTATTTCATTCCCAAAATG
qPCR-Solyc11g012980-F	ATGGTGAAGACAGAGCAAAAAAG
qPCR-Solyc11g012980-R	GCTGCAGCTTCAGGGGTAG
qPCR-Solyc06g007440-F	GGACATTAGAGATGGCCGTAG
qPCR-Solyc06g007440-R	ATTCGCCTCCTTTAACAAATTC
qPCR-Solyc04g050440-F	CATACCTCCTCTTCTCGGC
qPCR-Solyc04g050440-R	TTTTCCAATGAAACCATTAGAAGG
qPCR-Solyc10g083540-F	AGCCACTGCAAATTTCGAAGAG
qPCR-Solyc10g083540-R	CGTCTCTTCAGCTAATTCATATAC
qPCR-Solyc02g085020-F	CGCTACTGTTCGTGATCCTG
qPCR-Solyc02g085020-R	TTACTTCGTTCTCTGGATCC
qPCR-Solyc09g065100-F	AGAGAACTTTATGATTCACTTTCTG
qPCR-Solyc09g065100-R	TCATTTGCTCCCATTATCCAT
qPCR-Solyc09g074270-F	ATTTGATGTTGTTGATCGGTG
qPCR-Solyc09g074270-R	GACGACAGAATGTGTCGTAAAT
qPCR-Solyc08g066940-F	ATGGCAATTATTGAGGAGGAAG
qPCR-Solyc08g066940-R	CTTGTAGCTGTTGCGATTTC
qPCR-Solyc12g038160-F	ATGCAGCGGCTCGTTGAC
qPCR-Solyc12g038160-R	GGAAGCCTAGGCCCACG
qPCR-RNC1-F	TACTAAAACCCCCTTTAGATGATG
qPCR-RNC1-R	TCCTGACCTTACATGCCTAG
SlActin-qF	TGGTCGGAATGGGACAGAAG
SlActin-qR	CTCAGTCAGGAGAACAGGGT
